# An unusual condition during internal jugular vein catheterisation: vertebral artery catheterisation

**DOI:** 10.5830/CVJA-2016-040

**Published:** 2016

**Authors:** Ozge Korkmaz, Sabahattin Göksel, Öcal Berkan, Burçak Söylemez, Durmuş Kasım, Ahmet Cemil İşbir

**Affiliations:** Department of Cardiovascular surgery, Cumhuriyet university medical Faculty, Sivas, Turkey; Department of Cardiovascular surgery, Cumhuriyet university medical Faculty, Sivas, Turkey; Department of Cardiovascular surgery, Cumhuriyet university medical Faculty, Sivas, Turkey; Department of Neurosurgery, Cumhuriyet university medical Faculty, Sivas, Turkey; Department of head and Neck surgery, Cumhuriyet university medical Faculty, Sivas, Turkey; Department of Anaesthesilology and Reanimation, Cumhuriyet university medical Faculty, Sivas, Turkey

**Keywords:** vertebral artery, cannulation, internal jugular vein, anterior forminectomy, complication

## Abstract

Vertebral artery cannulation is an unusual complication during internal jugular vein cannulation. We report a case of vertebral artery cannulation, which occurred during an attempt to cannulate the right internal jugular vein, and we discuss the management of such a rare complication.

## Abstract

Internal jugular vein catheterisation is an essential procedure, specifically in major operations, such as cardiac surgery. Vertebral artery cannulation is a rare complication during internal jugular vein cannulation, but it may lead to sequelae that could prove fatal. When this condition occurs, it should be diagnosed quickly and treated as soon as possible.^1^

This case discusses a patient who had inadvertent vertebral artery cannulation during internal jugular vein access in the operating room for coronary bypass surgery. The right vertebral artery was repaired via foraminectomy using an intraoperative anterior approach, and coronary bypass surgery was subsequently continued.

## Case report

A 65-year-old male patient who, following diagnosis of coronary artery disease, had angiography and was admitted to the cardiovascular surgery unit for bypass grafting on four vessels. Carotid and vertebral artery Doppler ultrasonography, performed during pre-operative preparation of the patient, revealed no pathology in either the carotid or vertebral arteries.

After preparation, the patient was taken into the operating room for coronary bypass surgery. Peripheral venous and radial artery catheterisation was completed prior to general anesthaesia. Following the administration of general anaesthesia, the head of the patient was placed 15 degrees below the whole body, which was in the Trendelenburg position, and he was turned to the left. He was stained and covered under sterile conditions.

The puncture was made by an anaesthetist, using a 18-G needle, through the apex of the triangle composed of the sternal and clavicular parts of the sternocleidomastoid muscle. During puncture, the blood was established not to be bright in colour or pulsatile, and a 12-F × 15-cm double-lumen catheter was inserted using the Seldinger method. However, the blood from the catheter was bright and pulsatile, and so the catheter tip was attached to a transducer. From the arterial trace and the arterial nature of the blood sample taken, it was assumed that carotid artery catheterisation had been performed.

Doppler ultrasonography was carried out in the operating room on the patient under general anaesthesia. It was observed that the catheter was not in the carotid artery, and therefore the carotid artery was accessed by surgical exploration through an incision at the catheter tip. It was established from ultrasonography that the catheter had advanced deeper towards the cervical vertebrae.

In the ensuing minutes, brain, and head and neck surgeons were invited to the operating room. With neck dissection, the anterolateral view of the C5–C6–C7 vertebrae was accessed. It was seen that the catheter had entered through the C5–C6 disc space and had gone into the C6 vertebral foramen. The catheter was found to be in the subclavian artery of C7.

**Fig. 1:**
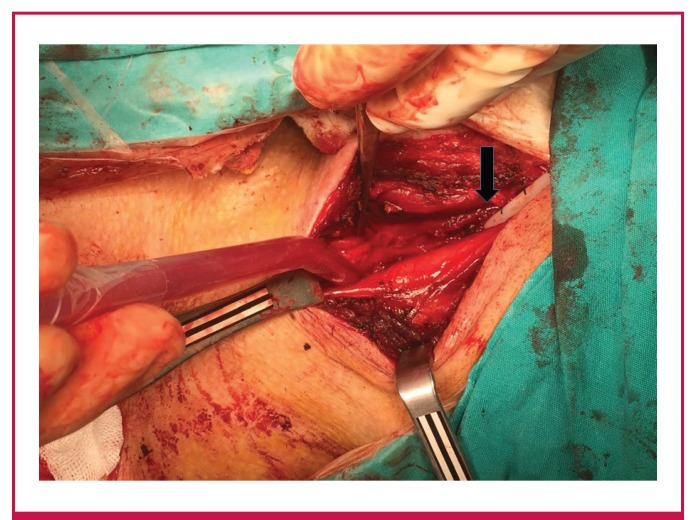
After foraminectomy the catheter was clearly seen advancing through the vertebral artery. The black arrow shows the side of the catheter.

Foraminectomy was performed anterior to the foramen of the C6 vertebra. This region was cleared and it was established that the catheter had advanced through the vertebral artery and was in the subclavian artery (Fig. 1). The catheter was withdrawn in a controlled manner, the existing vascular damage was primarily repaired, and coronary bypass surgery was then initiated.

During the operation, neurological soundness of the patient was followed up by the anaesthetists (pupil reflexes, absence of anisocoria, etc). The operation was completed without any problems and the patient was taken to the intensive care unit. He was awake and conscious at about postoperative hour four. A subsequent neurological examination revealed no pathology and the patient was extubated.

Tomographic angiography performed postoperatively revealed that the right vertebral artery was present at the outlet but totally occluded from about 0.5 cm (Fig. 2). The patient was again examined neurologically but no neurological deficit was identified. It was therefore recommended that he continue his treatment of low-molecular-weight heparin. His cardiac medication and other medical treatment were planned and he was discharged on condition of follow-up visits.

## Discussion

Internal jugular vein catheterisation is preferred in patients undergoing open-heart surgery, since it is easy to cannulate and is associated with a reduced risk of complications during cannulation; it is also distant from the surgical site. The prevalence of vertebral artery cannulation during internal jugular vein catheterisation is unknown, probably because there are fewer cases reported than actually occur. When we reviewed the literature, it was found to be reported less often than carotid artery punctures. Carotid artery puncture during internal jugular vein cannulation has been reported at a rate of 0.5 to 11.4%, whereas the rate of vertebral artery cannulation was from 0.099 to 0.775%,^2^ and fewer than 30 cases were reported on iatrogenic vertebral artery cannulation.^3^

Dissection, thrombosis, formation of arteriovenous fistulae, and pseudo-aneurysms are complications of vertebral artery injury during vein cannulation.^3^ Diagnosis of pseudo-aneurysm of the vertebral artery is often delayed because symptoms occur only late after cannulation.^4^ In particular, some patients may develop fatal vertebrobasilar ischaemia due to vertebral artery cannulation and associated severe and damaging sequelae, such as stroke or visual defects, while others may be asymptomatic, which can be attributed to sufficient extracranial collateral circulation.^5^

The best way to avoid iatrogenic vertebral artery cannulation is to take the necessary precautions. In other words, the best way to perform this procedure is under the guidance of ultrasonography, as recommended in many guidelines. However, this is almost impossible in emergent cases and when ultrasonography is not available or is difficult to access. In such cases, intervention may be performed using anatomical reference points.

The vertebral artery is classically the first branch of the ipsilateral subclavian artery, and arises from the posterior– superior part of this artery. The vertebral artery, after separating from the subclavian artery, generally passes through the transverse process of the C7 vertebra and superiomedially enters the transverse foramen of the cervical vertebrae (C6 in 95% of cases), extending vertically to the level of the C2 vertebra within the transverse foramen of the vertebrae.^6^ The extraforaminal region is about 4 cm and located deeper and more medially than the internal jugular vein. It is the region most open to injury, and in this region it is difficult to stop haemostasis with compression,^7^ because the vertebral artery courses in a deep plane.

A safer approach when arterial damage is caused, is with an immediate surgical or endovascular stent application by a wide-scale catheter, as stated by Hong-liang et al.^8^ These kinds of punctures frequently result from hyperextension of the neck, accompanied by excessive rotation of the head, or failure to adjust the direction and depth of the puncture, or using an excessively long needle to perform the puncture. In addition, the non-pulsatile flow from the first puncture, due to the thinner diameter of the vertebral artery, may be misleading. Both the quantity and colour of the flow are manifested more clearly when the final tip reaches the subclavian artery following catheterisation, as in our case.

## Conclusion

It should always be considered that during percutaneous interventions, vertebral artery cannulation may occur, even if there is only a slight probability. In the event of such a case, there should be a management plan, previously prepared by the surgeon and anaesthetists. If endovascular repair is not possible in vertebral artery catheterisations, or if the artery is completely occluded by the catheter, the catheter should be explored without delay, together with brain and/or head and neck surgeons, and the necessary repair procedure should be competently performed on the vertebral artery.
